# How Can Geoscience Communication Foster Public Engagement with Geoconservation?

**DOI:** 10.1007/s12371-023-00800-5

**Published:** 2023-02-20

**Authors:** Joana Rodrigues, Elsa Costa e Silva, Diamantino Insua Pereira

**Affiliations:** 1 Institute of Earth Sciences, Pole of the University of Minho, Braga, Portugal; 2 Communication and Society Research Centre, University of Minho, Braga, Portugal

**Keywords:** Geoscience communication, Public engagement, Geodiversity, Geoconservation

## Abstract

Geoscience and geodiversity, two sides of the same coin, deal with very poor social visibility and recognition. Ensuring the protection of geodiversity is not only in the geoscientists’ hands and all of society needs to be involved. Therefore, public engagement with geodiversity demands new solutions and a change of paradigm in geoscience communication. Most of the science communication activities undertaken by geoscientists, even when they use modern approaches and technologies, are mainly designed based on empirical experience, laid on didactical approaches and assuming the public’s knowledge deficit. In order to engage the society with geodiversity, it is not enough to focus on scientific literacy and deficit models in which lack of knowledge is considered to be the main obstacle between science and society. It is fundamental to establish a commitment between society and science based on dialogue where lay public is not seen anymore as a single entity with a knowledge deficit. Non-experts must become also protagonists in scientific decisions with social impact and integrate their knowledge and concerns in public participation and decision-making. Engagement with geoscience and geodiversity would benefit from more effective and targeted communication strategies, with different approaches to engage with communities, local stakeholders, media, students and teachers, scientific community, tourists, politicians or policy-makers, and groups with different concerns and distinct relations with science. In the last 20 years, science communication research has made many relevant contributions in order to promote more participatory processes with which society is asked to engage. Regarding geoscience communication as a discipline, it is a very recent Earth science branch that also incorporates social science, behavioral science, and science communication, but still lacks a clear and formal definition. This study provides a comprehensive review of the literature in order to develop a conceptual framework for geoscience communication research, identifying the main challenges and opportunities.

## Society and the “Invisible” Geosciences

Despite the progressive recognition of the importance of science in solving the main sustainability challenges of the planet Earth, geosciences are most of the time neglected (Brilha 2004; Prosser et al. 2010; Gordon et al. 2012; Stewart and Nield 2013; Stewart and Gill 2017).

The impact of human activities and the vulnerability of humankind to natural hazards is increasing as the world population keeps growing (Eder et al. 2009). Also the ecosystem services provided by geodiversity still need to be widely recognized and included in management policies, and geoscience plays an important role addressing these gaps (Gray et al. 2013; Brilha et al. 2018). This wider approach may contribute to reach the Sustainable Development Goals (SGDs) proclaimed by the United Nations Agenda 2030 (Gill 2017; Gill and Smith 2021).

The role of geoscience in the society has been intensified due to current challenges such as energy transition, groundwater management, climate change, extreme natural events, urban planning, or protection of the natural heritage. The understanding of those issues implies not only geoscientific knowledge, but also a deep understanding of the political, social, and economic elements, and their impacts on each person’s life. Earth science research is increasingly dealing with the interaction of physical and social systems, and so, social factors need to be incorporated into “physical” models (Oreskes 2015). New research opportunities, strongly committed to a sustainable environment, need a closer engagement with decision makers (NRC 2012; Acocella 2015). Moreover, the effective participation of society in the search of solutions to these challenges demands for scientists, stakeholders and communities’ engagement.

These and other environmental geoscience topics are very often focused by the media, but mainly because of their disaster side, that rarely approach positive aspects of research or direct implications on people’s lives (Liverman and Jaramillo 2011). Liverman (2009) argued that scientific communication is one of the main barriers to a proper use geoscience’s information in planning and in policy-making and that geoscientists need to become more and more involved in these processes. In addition, Broome (2005, p. 51) claimed that the “geoscience community has done a poor job of communicating its relevance to modern society”, thus affecting the social visibility and public perception of geosciences.

This understanding was, for example, behind the International Year of Planet Earth (2007–2009), promoted jointly by the International Union of Geological Sciences (IUGS) and UNESCO. This worldwide endeavour had as an ultimate goal to raise public and political awareness for the Earth science potential for improving the quality of life and safeguarding the planet (Mulder et al. 2006). The initiative, under the moto “Earth Sciences for Society,” was designed with a robust outreach program to generate interest and greater awareness among lay public as well as decision makers and politicians (Mulder et al. 2006).

Also new keywords in geosciences such as geodiversity, geoconservation, geoheritage, and geotourism with impact in daily life are growing and should be brought to the public (Thomas 2016).

Geoscience communication is a recent scientific field and appears as a challenge to address geoenvironmental growing concerns (Illingworth et al. 2018). Communication has been attracting the attention of geoscientists and practitioners who promote activities with the public, and share their experiences in scientific papers. However, few researches have framed the topic in broad and theoretical perspectives and considered the science communication research agenda. There is a clear gap between geoscientists, communicators, and science communication academic research and practitioners.

Being an emerging topic, geoscience communication needs a deeper conceptualization that could help to identify a research agenda and to improve geoscientists’ communication practices. The purpose of this article is to discuss the importance of science communication in the context of geosciences namely: (i) to provide an overview of the conceptual framework and current state-of-the-art, (ii) to systematize the concepts and models, discussing opportunities for the geosciences, (iii) to identify communication agents and practices, and (iv) to reflect about geoscience communication challenges, main debates, and gaps in the research agenda. Beginning with an outline of communication science models and current understandings of the needed relationship with the public, the article then builds on the emergence of the geoscience communication field and demonstration of its role regarding, geodiversity, nature conservation, environmental protection, and the planet sustainability. Examples of geoconservation communication current practices and of approaches engaging with geodiversity are presented. The article continues with the identification of existing challenges to science communication and finally discusses some pitfalls in the field and suggests guidelines for action.

## Communicating Science: Taking Science to Public

Communication between the scientific community and the public dates back to the seventeenth century. A debate about naming the Devonian geological period, in the 1830s and 1840s, occurred in pages of the popular magazine “The Athenaeum,” including scientific arguments with detailed stratigraphic sections (Rudwick 1985). Even so, only in the twentieth century the concern about the public’s knowledge emerged, considering how science and technology related with the awareness of their impact on society and policy choices (Gregory and Miller 1998).

The term “science communication” is often used as a synonym for different related concepts, such as “scientific literacy,” “popularization,” “informal education,” “public understanding of science,” or “public awareness of science.” They are not synonymous, and although they have compatible goals and fine boundaries, each one reflects specific contexts and approaches (Burns et al. 2003; Bauer et al. 2007; Trench 2008; Brossard and Lewenstein 2009). Terminology in science communication has evolved over time in parallel with the evolution of communication models and paradigms, as it will be discussed briefly below.

Burns et al. (2003, p 191) summarized science communication as the use of appropriate skills, media, activities, and dialogue to produce personal responses to science, such as awareness, enjoyment, or other affective responses; interest, evidenced by voluntary engagement; opinions, forming, reforming, and confirming science-related attitudes; and understanding of contents, processes, and social factors. There are several definitions for science communication, and it can be considered an umbrella term. A traditional concept of science communication appeared as an attempt to address the public’s knowledge deficit, recognized by scientists, through the promotion of “Scientific Literacy” (Miller 1983; Durant 1994). The concept is based on the premise that in democracies, science policy decision-making depends on the level of scientific knowledge of the population (Miller 1983). Conceptual overviews over “scientific literacy” show that it has expanded, and besides scientific knowledge, it has incorporated skills and attitudes (e.g., Shen 1975; Miller 1983; Laugksch 2000; Singh and Singh 2016).

“Science outreach” is frequently described as educational linked activities where scientists communicate their research to lay audiences, focusing on pre-university students, with the main purpose of increasing their scientific literacy (Illingworth et al. 2015). Sharing the same objective, the term “popularization” refers to strategies that simplify technical information and make science accessible (Hilgartner 1990; Lewenstein 2008; Bucchi and Trench 2016). There are also nearly equivalent terms originated in other languages, such as vulgarization (Bucchi and Trench 2016).

Another term with a broad scope is “dissemination.” Commonly used by research projects and funding agencies, it means sharing research results in different contexts with different audiences of potential users (peers, industry or other commercial players, and policy makers), (Wilson et al. 2010; European Commission 2022). It can be broadly included in the knowledge deficit model, since its mainly purpose is to transfer research results from the academy to society (Fogg-Rogers et al. 2015).

This deficit-model concepts are based on the idea that the public’s lack of scientific knowledge is a barrier between science and society, in which communication is a unidirectional process of transference, transmission, translation, and dissemination of information with the ultimate of goal of disseminate science and educate the audience. In the 1980s, the publication of the Bodmer Report (Royal Society 1985) recognized the importance of the public’s attitudes towards science and technology, pointing the beginning of a new paradigm in science communication, with the model of public understanding of science (PUS). According to this report, the more society knows, more would people appreciate science and thus develop positive attitudes (Bauer 2009; Royal Society 1985). Afterwards, the PUS model was understood as a sophisticated version of the deficit model, still privileging experts’ knowledge and keeping ignoring individual responses to information, depending on each person’s cultural and social experiences (Lewenstein 2003). Also, the term “Public Awareness of Science” appeared to emphasize the development of positive attitudes towards science, but in the end, it was just as a component of PUS and scientific literacy (Burns et al. 2003). Despite many efforts made with PUS’s initiatives, no significant changes were detected in society’s relationship with science, and a substantial crisis on confidence was recognized (House of the Lords 2000). While several researches have shown that knowledge and attitude are not directly correlated variables, recent studies warn that the attitudes towards science combine knowledge and self-confidence in a nonlinear correlation; confidence grows faster than knowledge. High confidence combined with average knowledge can lead to less positive attitudes to science (Francisco and Gonçalves-Sá 2019). This issue became clear during the COVID-19 pandemic crisis and also shows the need to invest in other strategies rather than just knowledge and facts transmission, as will be further discussed.

It was necessary to develop a new perspective, based on bidirectional communication and social responsibility (Bucchi 2008). A new “Science in Society” trend started with the goal of “restoring” public trust in science (Trench 2008; Wynne 2006). These concerns led to the first Public Engagement Conference, in 2007, organized by the European Union, in Lisbon, with the clear purpose of bringing citizens closer to science (European Commission 2008). This important meeting defined another shifting point for a new paradigm of public engagement with science and technology (PEST). PEST establishes a commitment between society and science based on dialogue, where non-experts become protagonists in scientific decisions with social impact (Pitrelli 2003) and where non-experts integrate their knowledge in public participation and decision-making (Burns et al. 2003; Katz-Kimchi et al. 2011; Wilson and Willis 2004; Bucchi and Trench 2016).

After the dialogue model, new dimensions were considered, namely participatory mechanisms for deliberating on science (Bucchi and Trench 2016). Citizen science is an example of active collaboration between the public and scientists, fostering community-driven research. This strategy may include citizens formulating research questions and hypotheses, discussing methods, collecting data, interpreting results, and discussing findings and its implications. A comprehensive overview of the benefits and challenges revealed that policy makers working with citizen science sometimes miss the opportunity to promote an effective dialogue with the people (Hecker et al. 2019). The main criticism to this strategy is that in many cases, citizens contribute only as data collectors, without actually co-creating processes (e.g., Minkman et al. 2017).

The boundaries between these terms are blurred and are often used interchangeably, but they reflect an evolution in the models and paradigms of research and practice in science communication.

The shift of the “Science AND Society” paradigm into “Science IN Society” demands a new model of “co-operative research,” a process of scientific knowledge co-produced, in close engagement between researchers and non-researchers (Stirling 2006). Bucchi and Trench (2021) propose an inclusive definition of science communication as the social conversation around science, a non-purposive, participatory, interdisciplinary, and open-ended endeavour. This agenda for the broad science communication has to be adapted to each specialized field of knowledge, but different rhythms have been noted in different sciences which calls for specific approaches for each field. Along with the paradigm changes already discussed, the concept of “publics” has also been changing. The plural form “publics” is used to show that “lay public” is not a single entity (Bucchi and Trench 2016). Burns et al. 2003 identified the following groups: scientists, mediators, decision-makers, general public (meaning non-experts), attentive public, and interested public. Research has shown that what differentiates audiences is more than demographics, role or their level of scientific literacy. It is their interest, attention, willingness, and relationship to specific scientific topics; it is a matter of concern (Costa et al. 2002; Burns et al. 2003; Bucchi and Trench 2016; Stewart and Lewis 2017).

## Geoscience communication

### The emergence of a research field

“Geoscience Communication” can be summarized as a practice which seeks to communicate aspects of geoscience with a wider audience, with the aim of increasing attention, involvement, and public discussion of geoscientific results (Illingworth et al. 2018). It covers aspects of outreach, public engagement, widening participation, or knowledge exchange (Illingworth et al. 2018). As a discipline, an independent research field, it is a very recent branch of Earth sciences, without a clear and formal definition. It needs better formalization, with systematic methodologies, academic rigor, and longitudinal evaluation, incorporating social science, behavioral science, and science communication (Illingworth et al. 2018; Hillier et al. 2021). Geoscience communication research includes interdisciplinary topics like education, outreach, communication, engagement and science policy, as well as process and mechanisms of public engagement with geoscience (Hillier et al. 2021).

In 2008, the Geological Society of London published “Communicating Environmental Geoscience,” the first volume dedicated to the topic with case studies referring strategies and new methods to stimulate more effective communication (Liverman et al. 2008). In 2015, the special issue “Effective Science Communication and Education in Hydrology and Natural Hazards,” conjointly edited by the Natural Hazards and Earth System Sciences (NHESS) and Hydrology and Earth System Sciences (HESS) journals, reflected the strengthening of reflections and discussions on the topic, held during the European Geoscience Union 2015 General Assembly (Bogaard et al. 2015). Later, in 2018, the first dedicated journal appeared, the geoscience communication (GC), edited by the European Geosciences Union. The journal not only shares scientific knowledge as it also fosters the recognition of the importance of science communication under the scope of geoscience (Illingworth et al. 2018).

In the same year, the conference “Communicating Geoscience: Building Public Interest and Promoting Inclusive Dialogue”, held at the Geological Society of London, discussed some of the main concerns on the topic, illustrating the current challenges of geosciences communication (Gibson and Roberts 2018), that can be summarized in two main issues:

- How can geoscientists take their investigations to diverse audiences, discussing specific techniques and strategies (“how to talk”);
- Geoscience communication is more than effective transfer of information from scientist to audience (“how to listen”).

This second challenge introduced the idea of the co-production of geoscience communication, with dialogue-based practices in order to encourage the public engagement (Gibson and Roberts 2018). Geoscientists as a group need to accept that only by “working together with the public” they can engage society in a constructive participatory way that will make geoscience reach public respect (Rosenbaum and Culshaw 2003).

Among the wide range geoscience topics, dinosaurs, volcanoes, precious stones, or meteorites seem to attract public’s interest and attention. However, most public concerns and discussions about Earth seem to be related with environmental topics such as mining, natural hazards, landslides, floods, earthquakes and climate changes, topics that besides risk, uncertainty, and controversy have significant political contours. Other subjects, such as the history of the Earth or evolution, although contested by specific some groups and cause of misinformation and fake news, do not seem to attract as so much interest or media coverage.

### From Scientific Literacy to Public Engagement with Geoscience

The background of the communication of geoscientific content to lay audiences goes back to the history of geoscience itself. It was in the mid-ninetieth century, when science became more specialized and started to be made by professionals, that the relationship between scientists and non-specialists became more relevant (Gregory and Miller 1998).

Mineral and fossil collection have been captivating great attention from devoted amateurs for mineralogy, petrology, and paleontology for centuries. The appearance of natural history museums in the seventeenth century, resulted from the fascination about natural wonders collections, that sparked public curiosity. The great scientist Charles Darwin and his book “Origin of Species” (1859) brought discussions to the public about the geological record and fossil evidence as testimonies of his theory of evolution. The book was written for a non-specialist public, and it is one of the most influential books throughout the history of science. In the eighteenth and nineteenth centuries, literature and art in general used geological landscapes, not only as source of inspiration, but also as a tool to provoke the sense of wonder and engage wider audiences (O’Connor 2007; Dean 2007; Heringman 2004; Gordon 2012). Scientific societies were responsible for launching public lectures that attracted crowds, as recorded in a peculiar incident, in 1839, when a struggle for free tickets for a geology talk by Benjamin Silliman caused a broken window at the ticket’s office (Gregory and Miller 1998). The American National Parks Service, after the Second World War, took around ten million young people to visit geological wonders, every year (Pangborn 1959). In the 1950s, the Boy Scouts of America promoted several initiatives, such as “The biggest show on Earth,” and in 1957, an outreach program with 4000 geologists and 38,000 scouts (Pangborn 1959).

As we have already discussed above, several terms have been used as synonyms of science communication and also in geoscience context, like “popularization” (e.g., Jacobi et al. 2016; Mariotto and Venturini 2017), “dissemination” (Pinto et al. 2011), “divulgation” (Farabollini et al. 2013), “outreach” (e.g., Saltzman 2014), or “vulgarization” (e.g., Rassou et al. 2019). “Popularisation” is the term with the longest tradition on science communication (Bucchi and Trench 2016) and one of the most used regarding geoscience communication.

Institutional and collective efforts and initiatives to bring geoscience to the public are well known, always trying to increase the awareness about geoscience and its importance (e.g., Eerola 2017; Losantos et al. 1989; Chen and Yang 2017; Patnaik 2019). Entities like governmental and scientific institutions, science centres and museums, protected areas, Geoparks, tourist caves, and mines (eg. Mariotto and Venturini 2017; Pereira et al. 2004; Justice 2018; Relvas et al. 2014) are used to bring geosciences closer to the public, promoting fieldtrips, exhibitions, workshops, festivals, thematic days, or public lectures (eg. Buddington and Garver 2003). There are also modern approaches to new technologies with online resources, video games, or smartphone’s software (e.g., Gravina et al. 2017; Mani et al. 2016; Rapprich et al. 2017; Reynard et al. 2015; Pasquaré Mariotto and Bonali 2021).

Relevant endeavours communicating geoscientific knowledge have been made by the American National Science Foundation that promoted the Earth science literacy (ESL) initiative. The ELS initiative produced the Earth Science Literacy Principles (ESLPs), a document that outlines what citizens should know about Earth science (Earth Science Literacy Initiative 2009). It defines an “Earth-science-literate person” with nine “Big Ideas,” endorsed by multiple representatives of the geoscience research, government, and professional communities (Wysession et al. 2012). These Big Ideas include geoscience research processes, fundamental content-based themes (complex interacting systems, internal and external geodynamics, or Earth’s history), and humans and their interactions with Earth (such as land-use, hazards, or natural resources).

ESLPs reflects “an understanding of Earth’s influence on people and of people influence on Earth” (Earth Science Literacy Initiative 2009). This is the message to be carried to the public and the reason why the entire Earth science community needs to increase outreach activities (Wysession et al. 2012). The document has the implicit goal to promote citizens’ involvement in public discussions. However, ESLPs is mainly centered on geoscience contents, focusing on a deficit communication model, where lack of knowledge is (still) the dominant obstacle between science and society.

## Geoscience Communication - Tool for Sustainability

Geoscience research has direct implications to human health, safety, and economic well-being; even so, most people are unaware that when they access news about water, climate change, natural resources, disasters, nature conservation, etc., they are accessing geoscientific information. Non-experts are still far from understanding the geodiversity’s role in everyday life and its importance in reaching effective sustainability for the planet. Society engagement in pursuing solutions for Earth’s challenges is limited, and public discussions are confined to few specific groups.

### Seeking Public Engagement with Geodiversity

Important and promising sustainable development strategies and policies like the Rio Earth Summit commitments (1992), the Millennium Ecosystem Assessment (2005), the United Nations Agenda 2030 and its 17 Sustainable Development Goals (2015), or Paris Agreement (2016) are mainly focused on the biotic features.

Under the scope of nature conservation, geodiversity has always occupied a secondary place, when compared to biodiversity as also the promotion of geodiversity’s benefits in delivering ecosystem services has been neglected (Gray 2004, 2018; Gray et al. 2013; Brilha et al. 2018). For this reason, geoconservation has very low priority among broad society and decision-makers (Prosser et al. 2011).

Despite this very low public awareness, society benefits directly and indirectly from geodiversity. However, to reach effective sustainable development, geodiversity values need to be broadly understood and protected (Brilha et al. 2018). The proper management of geodiversity is also fundamental to reach most of the targets in the SGDs (Gill 2017; Gill and Bullough 2017).

According to the Millennium Ecosystem Assessment, there are four groups of services (Millennium Ecosystem Assessment 2005). The “supporting services” support natural environments and life conditions, such as habitats provision, soil development, or water availability. The “regulation services” are natural processes that regulate the environment and allow the existence of life and modern society, like carbon sequestration, soil erosion, water quality or flood, seismic, and other geohazards protection. The “provision services” include the natural materials like water, mineral, or energy resources. The “cultural services” include knowledge, educational, and recreation or spiritual experiences. Geodiversity contributes to ecosystem services, based on its scientific, educational, economic, cultural, and aesthetic values (Brilha et al. 2018). In what concerns geoconservation, geological heritage and geosites can deliver cultural services, providing data to develop scientific knowledge and to be used for science communication proposes, in educational and tourist contexts (Pereira 2017; Pereira et al. 2018). It is clear that geodiversity underpins and delivers many vital ecosystem services. It is also clear that geoscience can contribute to integrated environmental management that includes geodiversity in order to implement solutions to broader environmental, economic, and social solutions (Gray et al. 2013). A research conducted in a Czech geosite assessed the public opinion on geodiversity and geoconservation. The study showed that although the visitors agreed that geodiversity must be protected, they found that it is more important to protect biotic than abiotic elements (Kubalíková et al. 2021). These results obtained in a geological heritage site which can be explained with the predominant emphasis only on the living nature. Lack and inefficient communication can account for the secondary role attributed to geoconservation when compared with biodiversity conservation (Crofts 2019). As science communication can contribute to biodiversity conservation (Bickford et al. 2012; Nadkarni 2004), it should also be a tool for geoconservation, requiring local geoscience initiatives to boost public engagement (Larwood and Durham 2005; Stewart and Nield 2013).

An integrated geoconservation strategy, beside inventorying, evaluating, protecting, or monitoring, must include the interpretation and public communication of geoheritage (Brilha 2005). Communicating geoheritage, focused on the geological content of geosites but also on their value and relevance, providing a cultural significance, will raise awareness for its environmental role and will foster public engagement with its conservation (Worton 2008; Macadam 2018) and may also have the indirect impact of engaging society for geo-hazards and risk reduction (Farabollini et al. 2014).

Azman et al. (2010) showed that communities’ engagement in the decision-making of territories like the UNESCO Global Geoparks, which considered their real interests and needs, lead to better attitudes towards geoconservation. This approach helps, for example, to overcome the limitations that conservation strategies may impose. Local communities’ participation in heritage conservation (Halim et al. 2011; Worton 2008) highlights the importance and respect given to indigenous knowledge, recognizing the importance of their culture managing their regions.

Geoparks are recognized for bringing science and society closer together, but data on their specific impact in local community’s scientific literacy and engagement in scientific participatory processes is still scarce and difficult to interpret and correlate. For example, a research conducted at a newly established Geopark and at a longer established Geopark indicated that visitors to the longer established Geopark do not necessarily have a greater awareness or knowledge of the Geopark or geology (Buhay and Best 2015). In 2021, the International Geodiversity Day was approved by UNESCO Executive Board to join world efforts raising awareness of geodiversity’s importance and role for society. In 2022, the date was celebrated for the first time on October 6th in an unprecedented global initiative, engaging society with geodiversity, through hundreds of worldwide activities, such as field trips, public talks, exhibitions, or publications.

### Geoconservation Communication Practices

According to Burek (2012), the awareness on geoconservation can be raised through geodiversity outreach and an increase in geotourism. Geotourism is, in fact, a relatively recent concept that has innovated the relationship between the public and geodiversity. It is a new tourism niche focused on geology and landscape (Dowling and Newsome 2006), involving all the conventional aspects of tourism activity. The focus on geology is supported by interpretive and service facilities that provide the understanding of geological sites (Hose 1998). Geotourism promotes geodiversity and geoconservation through appreciation and learning (Newsome and Dowling 2010; Allan 2015). The aesthetic value of a geosite and its tourist use may also foster its economic value (Gray et al. 2013).

The challenges of geoscience communication in geotourism are related with the nature of the geological phenomenon, often imperceptible to human senses, but also with the recreational element of tourism activities, too focused on subjective and aesthetic approaches (Garofano 2012). However, fascination with spectacular landscapes, the sense of wonder and the connection between geodiversity and cultural features can provide deeper awareness to geodiversity (Gordon et al. 2004; Gordon 2012). Also, TV fiction series, filmed in outstanding geological scenarios can engage the public in geoheritage (Lugeri et al. 2015). Outreach can be a first step to achieving understanding and appreciation for the conservation and protection of geological heritage (Burek 2012).

Geological heritage communication practices for lay public include in situ interpretation, like panels (e.g., Moreira 2012; Bruno and Wallace 2019; Mansur and Silva 2011; Macadam 2018), museums (e.g., Reis et al. 2014; Van Geert 2019), interactive exhibitions (e.g., Venturini and Mariotto 2019), geotrails, books, brochures and maps (e.g., Bartuś 2015; Bailey et al. 2007; Rodrigues and Neto de Carvalho 2009), TV series (Lugeri et al. 2015), guided visits, virtual reality tools (Tibaldi et al. 2020), or virtual tours (e.g., Martínez-Graña et al. 2013).

Virtual reality techniques raise new opportunities to communicate geoheritage, making geosites worldwide remotely available, providing immersive experiences in less accessible places and in sites with active geologic processes (e.g., Pasquaré Mariotto et al. 2020). These tools contribute to overcome some obstacles to the communication of geosciences, related to the abstract processes, time scales, or to the nature and complexity of the geological phenomena. They also acquired special importance during the restrictions in the context of the COVID-19 pandemic, creating alternative solutions to students and lay public to engage with geodiversity (e.g., Rader et al. 2021; Needle et al. 2022).

Most of the examples presented in this section sustain the prevalent didactic approach to geoheritage (Tormey 2020; Serrano and Trueba 2011; Mansur 2009), since the main goal is to increase the geological knowledge.

## Geoscience Communication Challenges

The main challenges of science communication are identified in the literature (e.g., Bucchi 2017; Ridgway et al. 2020), and they were intensified during the COVID-19 pandemic (e.g., Antiochou 2021; Rousseau 2021; Scheufele et al. 2021; Tennant et al. 2020). Geosciences, like any other scientific areas, have their specificities that influence communication, related to its conceptual nature, research methodologies, study subjects, and impacts on society, systematically reviewed below.

### Geoscientists Perceptions

Under the scope of the Communicating Environmental Geosciences working group of the IUGS Commission for “Geoscience for Environmental Management,” an international survey was conducted to assess about environmental geoscientist’s attitudes and experiences (Liverman and Jaramillo 2011). The results of this first approach to study the geoscientists’ community showed that these scientists were feeling well equipped to engage the public, although they seemed aware of the problems associated with communication. Despite motivation and perceived confidence about their skills and competences to communicate, geoscientists seem still very focused on unidirectional models and in communicating with their peers (Rodrigues et al. 2021).

Although most of them believe their subject is not too complex or difficult for the public to understand and to be covered by media, geoscientists admit some difficulties in the relation with media (Liverman and Jaramillo 2011).

The general barriers in effective geosciences communication, already identified for other fields, are related to the use of complex language, with the dissemination of results mostly in traditional scientific media, the identification of the target audience, the inaptitude to adapt products to diverse audiences, and lack of institutional support (Liverman 2009).

Constraints in communicating geoscience constitute an obstacle to develop new geological technologies for society, related, for example, to topics such as energy, mineral resources, or water (Gibson and Roberts 2018). Yehl (2016) argued that communication (together with commitment and collaboration) is one of the backbones of geoscience. Geoscience communication is not only performed by geoscientists, but also by other mediators such as journalists, teachers, science communicators, or geotourism guides. But due to the considerable role of geosciences in society, communication must be a fundamental activity for geoscientists (Martin and Peppoloni 2017); and despite the different conditions, practices, or motivations, geoscience communication became one of the everyday duties of many geoscientists (Illingworth et al. 2018).

### Geoscience Nature and Conceptual Constrains

Beside language and speech, geoscience communication faces significant conceptual challenges related with the complexity and multiplicity of Earth systems (Stillings 2012), contemporary environmental changes (Anderson and Brown 2010) or abstract, and not human-time scales (Kastens and Mandua 2012; Bowring 2014; Jacobi et al. 2016). Gaps in perception, related with different cognitive frameworks between geoscientists and non-experts, need to be considered, as shown by a study based on the mental models approach, that examined public perceptions regarding geological surface (Gibson 2017).

### Science Communication Training

General geoscience education prepares scientists poorly for science communication, public engagement, or communication with media. This topics should be included and reinforced in geosciences curriculums (Liverman and Jaramillo 2011; Ickert and Stewart 2016). Also, the scientific institutions play an ultimate role to support geoscientists: providing training, ensuring access to communication professionals, rewarding efforts to engage the public, and allocating adequate budget and staff (Liverman and Jaramillo 2011). For example, science communication training for geoscience graduation students, designed to answer specific challenges such as risk and emergency response, proved to be very effective improving students’ communication skills (Dohaney et al. 2015).

### Geoscience and the Policy-Making

Geoscientists feel their research is not always used effectively in developing policy, not only because of the lack of scientific background of policy-makers but also because scientists showed a poor understanding of the policy-making structures and process (Liverman and Jaramillo 2011; Simpson 2008; Petterson et al. 2008). More attention should be paid to the communication with decision makers, management, and planning authorities (e.g., Walsby 2008; Forster and Freeborough 2006; Kirchhoff et al. 2013).

### Risk and Uncertainty Communication

The management of the public health crisis during COVID-19 pandemic highlighted the discussion about ways to communicate risk and uncertainty, without creating excessive fear and even panic (Antiochou 2021). The decision-making processes under these conditions, with ideological and political constraints, and the importance of transparency were also discussed (Antiochou 2021; Scheufele et al. 2021). Public’s distrust on experts, polarized discussion, and politicized digital age amplified the challenges that society faces (Rousseau 2021). Research showed that trust in science was variable throughout the pandemic. In some moments, general trust has increased, but in others, space for misinformation and discredit appeared, these variations being closely related to political expectations (Bromme et al. 2022). These results reinforce the need to involve citizens in discussions about the process and contexts of knowledge production, including its uncertainties (Bromme et al. 2022).

These concerns are not new for geoscientists and have been discussed over time also regarding geological hazards and climate change. For geoscientists working on hazards, there are particular elements such as risk, probability, and uncertainty, which presuppose even more specific training, not only in basic communication skills but also in risk communication and media relations (Liverman 2009). Media tend to prefer the catastrophic and dramatic impacts of hazards, rather than scientific knowledge. The analysis of the extensive European and US press coverage of the ash impacts of the 2010 Eyjafjallajökull eruption showed that the amount of information available was high, focusing mainly on social themes, followed by volcanological, economic (17%) response, and airline issues (Harris et al. 2012). Volcanological information was usually placed down the reporting order; volcanologists made up 9% of the quoted sources, and negative words such as “stranded” or “chaos” were dominating (Harris et al. 2012).

Frequently, the communication of controversial themes leads to misinterpretation about research results, selectively used or quoted to advance policies or political objectives (Boykoff 2008) as, for example, the case registered in German media about carbon capture and storage research (Schneider 2019). The great polarization and the heated debates around topics like mining exploitation or climate change, in a “battlefield” mode, discourage many scientists from engaging with the media (Boykoff 2008). Regarding climate change, uncertainty is one of the main challenges especially when the aim is more than just to inform (Ward 2008; Schmidt-Thomé and Kaulbarsz 2008; Moser and Dilling 2012). Inadequate communication on geohazards, as earthquakes, volcanic activity, landslides or floods, can potentially have very serious consequences, with material and human losses (e.g., Sturloni 2012; Herovic et al. 2014; Benessia and Marchi 2017). Concerning seismic risk communication, it tends to be mainly focused on the conveyance of “geofacts” although these facts are closely linked with social issues of concern (Stewart and Nield 2013). In these contexts, strategies integrating local perspectives and social, political, and cultural concerns of communities influence positively communication efficiency (Ickert and Stewart 2016).

The lack of effective communication strategies clearly structured can result in poor communication and cause unwanted results, as seen in sensitive areas such as mining areas with environmental problems (Di Giulio et al. 2008). Miscomprehensions and cross-purposes can generate sensationalisms or political advantages and still push away the interested part (Schmidt-Thomé and Kaulbarsz 2008).

With the significant growth of geoenvironmental concerns, scientists face the major challenge of knowing how to communicate effectively such a complex body of knowledge (Illingworth et al. 2018). In order to address these challenges, geoscientists need to understand that objection and controversy are not merely a factor of bad communication (Gibson and Roberts 2018).

## Discussion and Conclusions

Geosciences face important obstacles when it comes to assert themselves in the public space: the field has poor public visibility, deficient mediatization, low impact in policymaking, and little engagement with the average citizen. This is the result, in part, of deficient science communication strategies.

When compared with broad science communication research, geoscience communication begins to affirm itself with a certain delay. The abundance of literature on effective science communication (e.g., Trench et al. 2014; Weigold 2001) still has little impact/relevance on geosciences. Despite the little research and innovation in this field, there are relevant exceptions such as the studies of Forster and Freeborough 2006, Liverman et al. 2008, Stewart and Nield 2013 or Drake et al. 2013, among few others.

A significant part of the geoscientific community and a large number of institutions already understand the importance of communication. Significant endeavours are done everywhere and, in many places, like museums, protected areas or UNESCO Global Geoparks (UGGps); communication with lay audiences is indeed a priority. However, most of these communication activities, as we have demonstrated in the literature review, even when using modern approaches and technologies, are designed based on empirical experience and assuming a public’s knowledge deficit. On one hand, geoscientists see the non-expert publics as students with insufficient knowledge and, on the other hand, geoscience communication strategies are often developed as educational activities (formal and non-formal), using common strategies and tools, as shown, for example, by the approaches of Mariotto and Venturini (2017), Mansur (2009), or Tormey (2020). Attached to this educational approach, scientists also tend to assume that if they can communicate the “facts,” the audience would then think like them, which does not happen, and countless geoscientists experienced situations where communication generated disturbance and increased apprehension (Gibson and Roberts 2018).

The communication paradigm for geoscience needs to be rethought and changed, from singular focus on public knowledge deficits as the guilty part, the solution for conflicts over science in society. It needs new ways of conversation and engagement that recognize, respect, and incorporate differences in knowledge, values, perspectives and goals, as it has been foreseen by science communication scholars, such as Nisbet and Scheufele (2009). A comprehensive framework for geoscience communication should be adopted, incorporating solutions, as some of those proposed by Drake and co-authors (2014), based on dialogue and engagement but also challenging the “matters of fact” paradigm by considering “matters of concern” approaches (Stewart and Lewis 2017). This framework should also consider new ways of engagement in order to include popular entertainment to reach larger audiences (Stewart and Nield 2013; Hut et al. 2016), as well as citizen science projects, for example, the monitoring of hazards such as mass movements, cliffs erosion, or sea level and coast lines. These projects boost public participation, where citizens participate actively as data collectors, even though this actual co-creation process is still an uncommon reality (Minkman et al. 2017). Strategies such as field-provoked communication workshops, community-centered participatory knowledge exchanges, and self-reflection on disciplinary practices and paradigms must be adopted and also incorporated into geosciences communication training, as suggested by Ickert and Stewart (2016).

In re-purposing geoscience communications, Stewart and Hurth (2021) propose a marketing-led approach of science communication—“guide-and-co-create,” to face long-term geo-environmental concerns. This purpose-driven, interdisciplinary, participatory, and reflexive model, focused on the well-being while co-creating the path to reach it, requires re-thinking the communication practices and changing geoscientist’s research practices (Stewart and Hurth 2021). This marketing approach for geoscience communication clearly meets the paradigm of co-operative research, engaging experts and non-experts in a social conversation, to co-create the paths to reach long-term beneficial outcomes, and should be reflected in a geocommunication framework. Another challenge identified in the review performed above is the communication of controversial themes, that can lead to misinterpretation. Scientists must oppose misinformation, in a geoethical attitude, conveying reliable scientific information fundamental to minimize impacts and, in the end, be able to prevent loss of human lives (Martin and Peppoloni 2017). Public is looking for prediction, anticipating events with details on place, time, or magnitude, and that is the reason that the nature of these research areas needs to brought to the public (Rosenbaum and Culshaw 2003).The fight against misinformation cannot only be carried out by conveying scientific facts and it has to include the audiences’ perspectives and legitimate concerns.

Geoscientists, stakeholders, social scientists, media, and society must establish the boundaries of their roles for an ethical communication (Solarino 2014), and this is a dimension that should also be considered in a comprehensive framework for geoscience communication.

In some specific contexts, such as geohazards, engagement strategies are being gradually adopted. Under the umbrella of the “International Strategy for Disaster Reduction” from United Nations, several national and international organizations and initiatives have been working for risk reduction, focused on a global approach, engaging individuals, and communities in how to manage hazards impacts (Eder et al. 2009). Local communities who live in geohazard areas benefit from effective and participatory communication, developing together strategies and measures to respond, preventing, and minimizing risks (e.g., Hermelin and Bedoya 2008).

Geoconservation practitioners, researchers, and all the geoscience community need to reflect and understand why geodiversity has so little public visibility and attention. Engagement with geodiversity and geoconservation forces to define targets and address specific strategies to specific audiences, understanding that “lay public” is not a single entity. As established in the literature on science communication, there are several publics, not only with different levels of knowledge, but mainly different groups with their own needs, interests, and cultural, social, and economic contexts (Costa et al. 2002; Burns et al. 2003; Bucchi and Trench 2016). This approach to public engagement should be incorporated in geoconservation communication. Thus, to engage the society with geodiversity, strategies should be targeted to different publics, such as local communities, local entrepreneurs and stakeholders, media, scientific community, tourists, politicians and policy-makers. In this setting, UNESCO Global Geoparks assume a relevant role engaging society with geoscience, geodiversity, and geoconservation. Given that the concept of Geopark combines protection and promotion of the geological heritage with sustainable local development (Eder and Patzak 1998; Zouros 2004), through bottom-up approaches and involving local communities (UNESCO 2015), UGGps have the proper setting and tools to be instrumental to bring these topics to public agenda.

The establishment of geoscience communication as a research discipline can benefit from following the methodologies and research agendas used by science communication scholars. With some exceptions, the geoscientific community tends to develop communication activities without logistic, financial, and technical support, based on empirical experience. In order to overcome this widespread limitation, cooperation with social scientists could help in the training of communication skills, in planning strategies to specific goals and audiences, and in dealing with media and with the challenging demands of a participatory model of science.

Geoscience communication as a discipline needs a better formalization, regarding its interdisciplinary approach and its specific challenges. In addition, the diversity and ambiguity of terms in the literature to refer science communication also reveals the immaturity of the topic. Communication strategies can put geosciences topics on the media and public agenda. To achieve this goal, communication practices must be targeted and change to an effective engagement paradigm, where society recognizes the importance of geoscience, geodiversity, and ecosystem services in planet Earth sustainability, and participate in their protection and management. Geoscience students and professionals would benefit from specific training on communication skills as well as on public engagement.

The comprehensive review of the literature contributed to identify the main challenges of geoscience communication. Most of the research and literature focus on specific activities and experiences, showing that geoscientists’ practices are far from science communication research and trends. Thus, to better understand the obstacles to geoscience communication, it is necessary to analyze the agents of geoscience communication. A close insight into geoscientists’ experiences and practices, as well as their motivations and perceptions, could better inform training policies and communication strategies and impact expectations. This further research may give us clues on how to improve geoscience communication, making it more frequent and more effective.
